# *Cannabis Sativa* Revisited—Crosstalk between microRNA Expression, Inflammation, Oxidative Stress, and Endocannabinoid Response System in Critically Ill Patients with Sepsis

**DOI:** 10.3390/cells9020307

**Published:** 2020-01-28

**Authors:** Anca Raluca Dinu, Alexandru Florin Rogobete, Tiberiu Bratu, Sonia Elena Popovici, Ovidiu Horea Bedreag, Marius Papurica, Lavinia Melania Bratu, Dorel Sandesc

**Affiliations:** 1 Faculty of Medicine, “Victor Babes” University of Medicine and Pharmacy, 300041 Timisoara, Romania; anca.dinu.umft@gmail.com (A.R.D.); alexandru.rogobete@umft.ro (A.F.R.); popovici.sonia@gmail.com (S.E.P.); marius.papurica@gmail.com (M.P.); lavi.ceuta@gmail.com (L.M.B.); dsandescumft@gmail.com (D.S.); 2 Clinic of Anaesthesia and Intensive Care, Emergency County Hospital “Pius Brinzeu”, 325100 Timisoara, Romania

**Keywords:** sepsis, endocannabinoid system, cannabinoids, cannabis sativa, THC, CBD

## Abstract

Critically ill patients with sepsis require a multidisciplinary approach, as this situation implies multiorgan distress, with most of the bodily biochemical and cellular systems being affected by the condition. Moreover, sepsis is characterized by a multitude of biochemical interactions and by dynamic changes of the immune system. At the moment, there is a gap in our understanding of the cellular, genetic, and molecular mechanisms involved in sepsis. One of the systems intensely studied in recent years is the endocannabinoid signaling pathway, as light was shed over a series of important interactions of cannabinoid receptors with biochemical pathways, specifically for sepsis. Furthermore, a series of important implications on inflammation and the immune system that are induced by the activity of cannabinoid receptors stimulated by the delta-9-tetrahydrocannabinol (Δ9-THC) and cannabidiol (CBD) have been noticed. One of the most important is their ability to reduce the biosynthesis of pro-inflammatory mediators and the modulation of immune mechanisms. Different studies have reported that cannabinoids can reduce oxidative stress at mitochondrial and cellular levels. The aim of this review paper was to present, in detail, the important mechanisms modulated by the endocannabinoid signaling pathway, as well as of the molecular and cellular links it has with sepsis. At the same time, we wish to present the possible implications of cannabinoids in the most important biological pathways involved in sepsis, such as inflammation, redox activity, immune system, and epigenetic expression.

## 1. Introduction

Sepsis is one of the main causes of death in the intensive care unit (ICU) worldwide. International statistics present significant results regarding the incidence of sepsis, with approximately 30 million people being affected by it, leading to death in over 6 million of these cases [1,2,3]. This can also be caused by bacterial, fungal, parasite, or viral infections, as well as different combinations involved in sepsis. Organ dysfunction seems to have a more pronounced biochemical substrate in comparison with the initial organic substrate, becoming a reversible affection, although the mortality risk remains high [4,5,6].

The complexity of these cases is given by both the molecular and initial cellular imbalances, as well as by the specific secondary responses [7,8,9]. The main mechanisms involved in the pathogenic cascade and in the evolution of sepsis are represented by the inflammatory response, immune response, and redox activity, followed by the involvement of cellular defects, which all lead to severe organ dysfunction. Adding to this phenomenon are severe respiratory infections; tissue hypoxia; acute kidney injury (AKI); metabolic imbalances; and, last but not least, derangements of the biological system as a whole. Through the augmentation of the inflammatory status and through the concomitant derangement of several organ functions, a high percentage of critical patients with sepsis develop multiple organ dysfunction syndrome (MODS), and finally death [8,10,11,12].

Numerous biochemical and cellular systems involved in sepsis pathogenesis have been studied, both in order to better understand these complex mechanisms, and in the hope of finding new therapeutic targets. The endocannabinoid response system is one of them. Recent studies have shown the involvement of specific endocannabinoid receptors, such as endocannabinoid CB1 receptor and CB2 receptor, as well as their link with important processes in sepsis, such as the immune response, inflammatory response, and redox activity [13]. Furthermore, a series of implications on the epigenetic processes have also been proven, through the change in the microRNAs expression, the microRNAs responsible for the modulation of the immune, and inflammatory systems [14]. It was also shown that by stimulating the CB1 and CB2 receptors through cannabinoids such as delta-9-tetrahydrocannabinol (Δ9-THC) and cannabidiol (CBD), there are important changes in the main biochemical and cellular mechanisms involved in sepsis [15]. Among these, we mention the effects on the inflammatory profile, in the immune response, on metabolism, and, on the metabolic status. Different research groups have also shown the impact of cannabinoids on the expression of microRNAs, and on the transcription mechanisms and genetic modulation of cellular processes [16]. 

The aim of this paper is to present, in detail, the biochemical and cellular processes involved in sepsis, and to find correlations between the mechanisms of the endocannabinoid signaling system and the pathophysiological mechanisms of sepsis. Furthermore, we wish to present and put into context the implications of endogenous and exogenous cannabinoids on the inflammatory and redox profiles, and on the immune response, in order to present as complete of an image as is possible of their involvement in sepsis.

## 2. Pathophysiological and Clinical Aspects in Sepsis

From a pathophysiological viewpoint, the first step in sepsis is characterized by the initiation of the immune response, represented by the activation of macrophages, neutrophils, monocytes, and natural killer cells [17,18,19]. The molecular and cellular process is modulated by the recognition of bacterial toxins or fungal beta-glycans on different receptors. Another molecular factor responsible for the activation of cells is represented by the damage-associated molecular patterns (DAMPs) [20,21,22]. DAMPs can be represented by the mitochondrial DNA, by ATP, or by molecular fragments released from the damaged cells. To be more exact, the activation of immune modulation signals is carried out through their simulative binding with specific receptors found on monocytes and macrophages, such as Toll-like receptors (TLRs) [23], nucleotide binding oligomerization domain (NOD-like receptors) C-type leptin receptors, and retinoic acid inducible gene 1 (RIG-1) receptors. Regarding the activity of B- and T-lymphocytes, it was proven that the antigen is recognized by T lymphocytes through the T-cell receptor. A very important aspect is that the CD4+ T helper cells use the major histocompatibility complex class II (MHC II) for antigen recognition [24,25,26]. Following the biomacromolecular and cellular reactions, increased amounts of proinflammatory cytokines are released, such as tumor necrosis factor alpha (TNF-α), interleukin-1 (IL-1), interleukin-1 beta (IL-1β), and interleukin-6 (IL-6) [27,28,29]. The released pro-inflammatory mediators enter a vicious circle, leading to the activation and proliferation of leucocytes, the overexpression of the endothelial adhesion molecules, the activation of the complement system, and to the overproduction of tissue factors and free radicals (FR) [30].

The augmentation of the inflammatory and oxidative status leads to the activation of the coagulation cascade. In this situation, microclots are being produced that are responsible for the blockages in the microvascular system, leading to tissue hypoxia and end-organ damage. This is mainly due to the production of thrombin and to platelet activation. From a clinical point of view, the accumulation of these molecular and cellular dysfunctions will lead to a series of phenomena, such as the overexpression of the inflammatory response, an increase in cell hypoxia, impact on metabolism, and an impact on the main biological systems. All of these together lead to the already known clinical response manifested as fever or hypothermia, hemodynamic changes with hypotension, respiratory distress, kidney injury, coagulation disorders, neurological decline, and acid–base imbalances [31]. An increased number of critically ill patients with sepsis also develop a series of secondary neurologic issues, such as confusion, agitation, lethargy, or disorientation. The pro-inflammatory and pro-oxidative model specific for these patients is characterized by the excess production of cytokines, and reactive oxygen and nitrogen species. Papadopoulos et al. have reported the fact that important pro-inflammatory mediators released by leukocytes in sepsis have a significant neurotoxic effect. They exert their toxic effect, especially on neurons, astrocytes, and endothelial cells. In other words, the biological effects are represented by the formation of chemotactic factors, by lipid and protein oxidation, the oxidative attack on DNA-structures, excessive production of tumor necrosis factor alpha (TNF-α), and an increase in the concentrations of peroxynitrite and interleukin 1 beta (IL-1β) [32]. Apoptosis is another widely studied phenomenon, both in the context of the immune system and in the context of other cellular systems such as neurons, epithelial cells, cardiac monocytes, and endothelial cells [33,34,35,36]. Together with the multicellular damage induced by sepsis, the augmentation of the pro-inflammatory and pro-oxidative status leads to a series of organ dysfunctions that, in turn, lead to multiple organ dysfunction syndrome (MODS), worsening of the clinical status, and finally death [18,37,38,39].

## 3. Biochemical Aspects of Cannabinoids 

Substances from the class of cannabinoids are found in high concentrations in the plant of *Cannabis sativa* [40]. Out of the many structural forms of cannabinoids, the most widely studied are delta-9-tetrahydrocannabinol (Δ9-THC) and cannabidiol (CBD) [41]. Nevertheless, there are over 100 molecular structures [13] existing as types of C-terpenopenols, which, based on their atomic dispositions and their macrostructures, have been classified as delta-8-tetrahydrocannabinol (Δ8-THC), Δ9-THC, CBD, and cannabicyclol. Moreover, based on their origin, they can be classified as phytocannabinoids, endocannabinoids, and synthetic cannabinoids [42]. The endocannabinoid system is involved in various processes, such as lipolysis, energy balance, metabolism, and cognition and behavior. The most important endocannabinoid system are the anandamide (AEA) and 2-arachidonoylglycerol (2-AG), which also have high concentrations in the brain [43,44,45]. 

**Figure 1:**
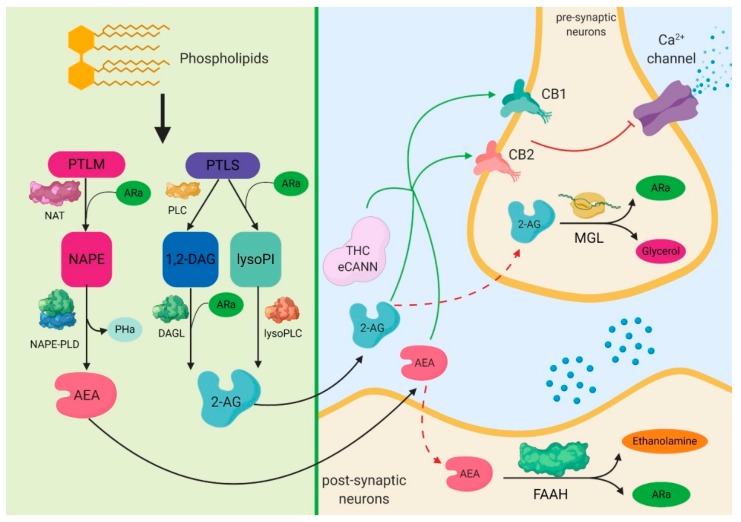
Molecular signaling mediated by the endocannabinoid system in the central nervous system. The most important endocannabinoid systems are the anandamide and the 2-arachidonoylglycerol, which also have high concentrations in the brain. Anandamide can be obtained through a series of biological mechanisms that involve the restructuring of phospholipids by the *N*-acyl-transferases. 2-arachidonoylglycerol results from the action of phospholipase C-beta enzymes or after the dephosphorylation of lysophosphatidic acid. Following these reactions, both anandamide and 2-arachidonoylglycerol activate the G-protein coupled receptors, transient receptor potential vanilloid type 1, and peroxisome proliferator-activated nuclear receptors. Finally, cyclooxygenase-2 can act on these two endocannabinoids through redox mechanisms, leading to the production of prostamides and prostaglandin-glycerol esters. PTLM: lipid precursor phosphatidylethanolamine; NAT: *N*-acyltransferase; ARa: arachidonic acid; NAPE: *N*-arachidonoyl-phosphatidylethonolamine; NAPE-PLD: NAPE-phospholipase D; PHa: phosphatidic acid; AEA: anandamide; PTLS: lipid precursor phosphatidylinositol; PLC: phospholipase C; 1,2-DAG: 1,2-diacylglycerol; lysoPI: 2-arachidonoyl-lysophospholipid; DAGL: diacylglycerol lipase; lysoPLC: lysophosphilipase C; 2-AG: 2-arachidonoylglycerol; CB1: cannabinoid receptor 1; CB2: cannabinoid receptor 2; THC: tetrahydrocannabinol; eCANN: endogenous and exogenous cannabinoids; MGL: monoacylglycerol lipase; FAAH: fatty-acid amide hydrolase.

From biochemical point of view the endogenous cannabinoids are obtained through the action of *N*-acyl-transferases on the endogenous phospholipids. The lipid precursor phosphatidylethanolamine (PTLM), through the action of *N*-acyltransferase (NAT) and of arachidonic acid (ARa), leads to the formation of *N*-arachidonoyl-phosphatidylethonolamine (NAPE), which, through the action of NAPE-phospholipase D (NAPE-PLD), forms *N*-Arachidonoylethanolamine (anandamide—AEA) and phosphatidic acid (PHa). There are two pathways regarding the mechanisms that lead to the formation of 2-arachidonoylglycerol (2-AG). One of them is represented by the phospholipase C (PLC), which acts on the lipid precursor phosphatidylinositol (PTLS) to form 1,2-diacylglycerol (1,2-DAG). Through the action of diacylglycerol lipase (DAGL) and arachidonic acid (ARa) on 1,2-DAG, 2-AG is formed. Another pathway is represented by the action of ARa and phospholipase A1 on phosphatidylinositol, which leads to the formation of 2-arachidonoyl-lysophospholipid (lysoPI). This further leads to the formation of 2-AG through the action of lysophosphilipase C (Lyso-PLC). The result of this activation of cannabinoid receptors (CB) by different molecules, including endogenous and exogenous cannabinoids (eCANN) such as tetrahydrocannabinol (THC) or cannabidiol (CBD), will act on the calcium channels in order to decrease and inhibit, at a pre-synaptic level, the influx of calcium, therefore inhibiting the release of glutamate or gamma aminobutyric acid (GABA). The inactivation of endocannabinoids takes place by their transport inside the cell and by hydrolysis. Two different enzymes are involved in this reaction, monoacylglycerol lipase (MGL) for 2-AG, and fatty-acid amide hydrolase (FAAH) for AEA. These have been targets in recent research on the treatment of pain, with pharmacologic inhibitors being studied for both the deactivation of the endocannabinoid action pathway and for their transport [45,46,47,48,49,50,51,52,53,54,55,56,57,58,59,60,61,62,63,64,65,66,67,68,69,70,71] (Figure 1).

The activity of cannabinoids depends solely on their interaction with certain cannabinoid receptors (CB), part of the G-protein coupled receptor family. CB1, CB2, and CB3 have been identified. From a biological point of view, CB1 can be found in the central nervous system [51,52,53]. Their share is higher in certain regions, such as the amygdala, cortex, hypothalamus, hippocampus, and the cerebellum. CB2 can be mainly found in the immune system, with higher concentrations in lymphocytes, macrophages, and inside the spleen. CB3 has been identified inside the endothelial cell and in the spleen [54]. Gαi is one of the most well understood biochemical pathways for the activation of CB1 and CB2 receptors, which also includes adenyl cyclase (Ac), mitogen activated protein kinase (MAPK), and cyclicadenosine monophosphate (cAMP) [46,55,56].

## 4. The Expression of Cannabinoid Signaling System in Sepsis

**Figure 2:**
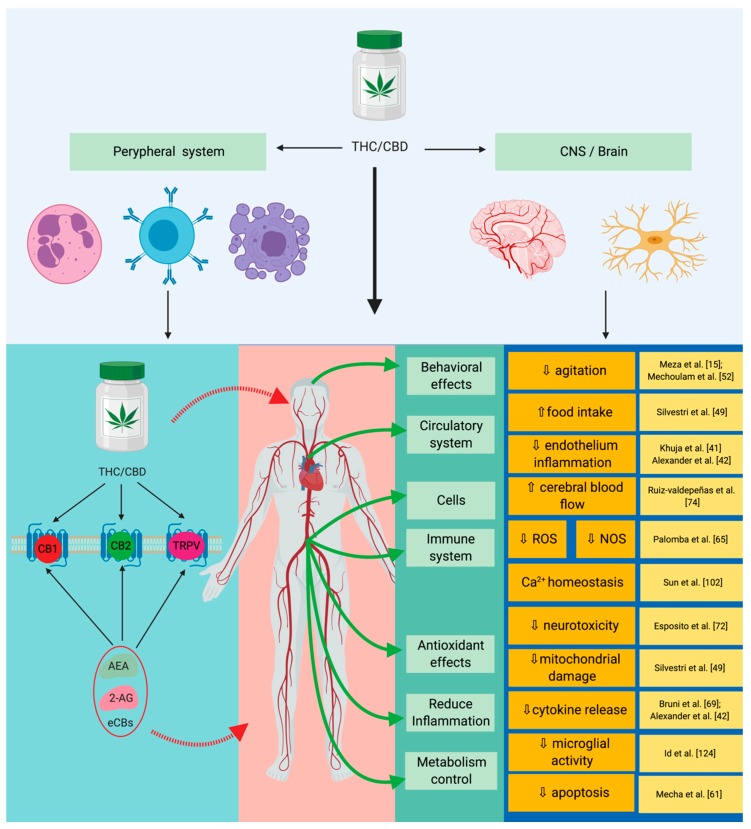
Implications of the endocannabinoid system in sepsis. By acting on specific receptors CBD/THC lead to the suppression of cytokine production, as well as to a reduction in redox mechanisms. Through their synergistic action these will lead to the reduction of the systemic inflammatory response and to an immune modulation with neuronal protection. THC: tetrahydrocannabinol; CBD: cannabidiol; CB1: cannabinoid receptor 1; CB2: cannabinoid receptor 2; TRPV: transient receptor potential vanilloid type 1; AEA: anandamide; 2-AG: 2-arachidonoylglycerol; eCBs: endogenous cannabinoids.

Recent studies have shown a series of anti-inflammatory, antioxidant, and antiapoptotic properties of cannabinoids in sepsis (Figure 2). In practice, the link between the cannabinoid system and sepsis can be described in a simplistic manner, through its effects on inflammation, on the immune system, and on the redox activity (Table 1) [15].

A very important, but also specific, aspect for the septic patient is malnutrition. It can be induced either by the specific hypermetabolism, or by the under-dosed nutrition of patients in the ICU. Moreover, in the same context, a phenomenon that impacts the quality of life is represented by the recovery period and by the reintegration of the former patient in the society [57,58,59,60].

Recent studies have concluded that endocannabinoids are characterized by a very fast response time to metabolic changes throughout the body. The activation of the CB1 receptor leads to the release of certain orexigenic peptides via neurons in the hypothalamus. What this means is that the stimulation of the CB1 receptor by Δ9-THC leads to an increased appetite for foods. These mechanisms are regulated by feeding-regulation hormones, such as orexigenic leptin, orexigenic ghrelin, and glucocorticoids. Another very important aspect regarding the impact of CB1 and CB2 receptors on the metabolism is given by the modulating properties of the CB1 on lypogenesis and on the control of the glucose levels. Some studies have shown that through the interaction of CB1 and 2-AG receptors, as well as with AES, one can increase the glucose metabolism, activate mitochondrial biosynthesis, and reduce adiponectin production [54,61,62].

## 5. The Cannabinoid Signaling System and Inflammation-Linked with Sepsis

Cassol-Ju et al. carried out a study regarding the effects of cannabinoids on the pro-oxidative profile in the brain and other organs, and on cognitive function in lab animals with induced sepsis. Following their study, they reported a significant decrease in the prooxidative and proinflammatory profiles. Moreover, they observed a decrease in the expression of thiobarbituric acid reactive species (TBARs) and of protein carbonyl in subjects that benefitted from CBD [76]. Furthermore, Cassol-Jr et al. also reported a decrease in mortality in subjects treated with CBD [76]. Another interesting study was carried out by Prester et al., regarding the proinflammatory changes induces by cancer therapies with Irinotecan, and the effects Δ9-THC has in this field. One of the already known effects of Irinotecan therapies is the increase of aspartate aminotransferase (AST). Following this study, they observed a significant decrease in the AST levels in the group that received double therapy with irinotecan and Δ9-THC [68]. Acute lung injury (ALI) and acute respiratory distress syndrome (ARDS) are two other pathologies frequently associated with sepsis, and yielding high mortality rates. From a morphopathological point of view, ALI and ARDS present similarities, such as the hyperinflammation of the lung tissues. From a clinical viewpoint, these contexts are associated with severe hypoxemia, leading to the impossibility of assuring effective ventilatory support. Regarding the mechanisms behind them, the causes can be divided into direct and indirect, such as lung injury, inhalation injury, pulmonary contusions, burns affecting the airway, disseminated or localized infections, and cellular lesions induced by massive blood transfusions. In the case of sepsis, the inflammatory profile is augmented by the recognition of certain bacterial antigens on the macrophage receptors, such as Toll-like receptors 2 and 4 (TLR2 and TLR4). This affects the integrity of the endothelial tissue, with the alveoli being exposed to a series of molecular and cellular attacks by fluids and macromolecules. Vuolo et al., in another study, focused on the evaluation of the anti-inflammatory activity of cannabinoids in asthma, have reported a decrease of IL-4, IL-5, IL-13, IL-6, and TNF-α. Following this study, the group highlighted the potential of cannabinoids to modulate the anti-inflammatory response in the context of asthma [77]. Chiurchiu et al., in a similar study, proved a decrease in the IL-6, IL-12, and IFN-α levels in dendritic cells [45]. In regard with the antimicrobial activity of *Cannabis sativa* extracts, studies have reported antimicrobial characteristics targeting gram-positive (*Staphylococcus aureus*, *Micrococcus flavus*, and *Baccilus subtilus*) and Gram-negative bacteria (*Proteus vulgaris* and *Pseudomonas savastanoe*), as well as an antifungal activity (*Aspergilus niger*) [40,78,79,80,81]. Appendino et al. also proved an increased antimicrobial activity for Δ9-THC and CBD on *Staphylococcus aureus* [82]. A similar study was carried out by Bass et al., who reported a significant decrease in neurological damage inflicted by infections with *Streptococcus pneumoniae* following long-term CBD administration [83]. Finally, Chakraborty et al. reported the beneficial antimicrobial effect of *Cannabis sativa* on methicillin-resistant *Staphylococcus aureus* [84]. A high percentage of critically ill patients with sepsis present a complete loss of appetite, as well as a reduction of metabolic activity. Recent studies have shown that by stimulating the CB1 receptor and the central modulation mechanisms, one can obtain a significant increase in voluntary food intake. Bellocchio et al. have shown, in an experimental study, that the administration of titrated doses of CB1 receptor agonists significantly increases appetite. On the other hand, the same group has also proven that by increasing the CB1 receptor agonist doses, there is a sudden decline in feeding in lab animals [85]. Sardinha et al. carried out a study on the CB2 receptor modulation under conditions of lipopolysachharide (LPS)-induced sepsis. Following this study, they observed a decrease in both the leukocyte expression and endothelial interactions. For this study, the group used different cannabinoids, reporting a decrease in the number of adherent leukocytes induced by the HU-308-CB2 receptor agonist [86]. 

Another complex study regarding the modulation of the inflammatory response induced by CB2 receptor activation was carried out by Wang et al. on skin wound healing [87]. For CB2 activation, they used extremely specific agonists and antagonists for cannabinoid receptors, such as GP1a [1-(2,4-dichlorophenyl)-6-methyl-*N*-piperidin-1-yl-4*H*-indeno-[1,2-c]pyrazole-3-carboxamide], and AM-630 [6-iodo-2-methyl-1-(2-morpholin-4-ylethyl)indol-3-yl-(4-methoxyphenyl)methanone]. Following the activation of the CB2 receptor, they observed a decrease in the expressions of IL-6, IL-1β, TNF-α, vascular endothelial growth factor (VEGF)-A, stromal cell-derived factor (SDF)-1, and monocyte chemotactic protein (MCP)-1 [87]. Chiurchiu et al., in a study on the AEA endocannabinoid effects on primary human T-lymphocytes, showed a strong immunomodulatory effect on the cellular function. Furthermore, they highlighted a reduction in cell proliferation induced by AEA in this particular context. Last but not least, an important decrease in AEA levels has also been proven in this context, as well as a decrease in IFN-γ and TNF-α from CD8^+^ T cells [43]. Eisenstein et al. also reported a reduction in the primary and secondary antibody production in splenocytes, as well as a decrease for the Th2 activity in human T cells [88]. Regarding the specific inflammation characteristics for lung injuries, Ribeiro et al., observed a reduction in IL-6, MCP-1, TNF-α, and MIP-2 expressions following the administration of a single dose of CBD (20 mg/kg) [89].

## 6. Cannabinoid Signaling System and Redox Activity-Linked with Sepsis

One of the most aggressive molecule structures responsible for the augmentation of inflammation is represented by atoms with a deficit of electronic stability that are called free radicals (FRs). FRs are produced under normal metabolic conditions, with their activation being normally inhibited by a series of endogenous and exogenous antioxidant substrates. In hypermetabolic states like inflammation and sepsis, the oxidant/antioxidant balance is significantly disrupted, with an exaggerated increase in FR expressions. This major imbalance that becomes systemic in a very short period of time is called oxidative stress (OS), and is involved in the majority of biochemical pathways [90,91,92,93,94,95]. 

In recent last years, academia has brought great focus on the redox expression in critically ill patients, such as an FR attack, and modified a series of biological systems, such as the mitochondrial DNA, the structure of lipids, the structure and activity of proteins, and the cytoplasmic systems. If we look at the overall context of sepsis, macrophages and neutrophils are the main substrates responsible for increased FR, reactive oxygen species (ROS), and reactive nitrogen species (RNS), affecting important processes such as cellular metabolism, angiogenesis, and the mitochondrial respiratory chain [96]. Under prooxidative and proinflammatory conditions specific for critically ill patients with sepsis, a disrupted mitochondrial activity leads to major energy deficits, therefore increasing the production of new inflammatory mediators, and in the end leading to clinical complications associated with sepsis [91,97,98]. This fact becomes extremely important as the mitochondrion represents the “power engine” of the cell, directly responsible for energy production under the form of adenosine triphosphate (ATP). The mitochondrial genome will be affected during the redox stress specific for sepsis, and during increasing the inflammatory response. Structurally, the mitochondrial DNA (mtDNA) contains approximately 16 kilobases, and is responsible for coding certain specific and essential processes involved in the metabolic functions of the cell. Among these is the modulation of 13 genes from the respiratory chain, I, III, IV, and V, as well as 22 genes responsible for the expression of the mitochondrial tRNA [21,99,100]. mtDNA is extremely vulnerable to redox attack because of the limited DNA repair mechanisms, as well as the lack of adequate histone coating. In this critical situation, the mitochondrial activity is substantially affected by the alteration of the molecular mechanisms, and of the respiratory chain and by the imbalances in the energy pathways [101]. The specific reactions for redox imbalances are represented especially by the coupling of the superoxide anion with nitrogen monoxide, leading to the production of a series of extremely reactive molecular species, such as peroxynitrite. Other substrates with a strong oxidative character are represented by the hypochlorous acid and hypobromous acid resulting from the oxidant reactions of the hydrogen peroxidase. The attack of these free radicals on the mitochondrial structures first impacts on the respiratory chain and on the electron transport chain, leading to an accelerated increase in membrane permeability through the reduction of its electric potential. As a consequence, numerous fragments resulting from the mtDNA destruction will be released, which will further interact with the immune system. This cascade of molecular interactions will lead to the activation of macrophages, neutrophils, and TLR-cells, with a further augmentation of the inflammatory status and acceleration of the cellular redox stress [95,96,97,98,99,100,101]. 

Sun et al. investigated the neuroprotective effects of cannabinoids in the context of oxygen–glucose-deprivation/reperfusion. Following their study, they observed that cannabinoids have direct implications on minimizing OS and on reestablishing mitochondrial and glucose metabolism through the pentose-phosphate pathways [102]. Another effect on the molecular mechanisms of cannabinoids is represented by the reduction of the NO synthase protein (iNOS) activity, and by the inhibition of the TNF-α activity. Furthermore, they reported their involvement in the excessive transmembrane Ca^2+^, as well as in minimizing the enzymatic hydrolysis in the redox context. Burstein et al. reported a decrease in redox activity due to hypoxia and ischemia in newborns [103]. Carrier et al., in a similar study, showed an increase in the adenosine levels in the brain, leading to reduced inflammation-related neurotoxicity [104]. Castillo et al., after carrying out a similar study, reported a significant decrease in hypoxia-induced brain damage phenomena. The main segments involved are represented by the reduction in the expression of IL-6, TNF-α, and iNOS [105]. Moreover, they have shown that the neuroprotective mechanisms are mediated by the CB2 receptors and by adenosine through the A2A receptor [105]. Even more interesting is the fact that increased levels for CB1 receptors have been reported on the surface of the mitochondrial coating. Hebert-Chatelain et al. showed that CB1 receptors interact with the respiratory chain complex I, which is responsible for the modulation of the redox activity in this segment [106]. Palomba et al. carried out a study on CB1 receptors and on the redox modulation capacity in the arcuate nucleus neurons, and its interaction with the appetite-regulating hypothalamic neuropeptides. The group observed a decrease in leptin-mediated processes, with a significant reduction in the ROS production in hypothalamic neurons cellular lines. Furthermore, they observed that in these redox processes, blocking mechanisms intervene in the activation of catalase and peroxisome proliferator-activated receptors gamma (PPAR-γ) [65]. Under the critical conditions expressed in sepsis, due to accentuated inflammation, and because of accelerated redox processes, there is an overexpression of neuronal inflammation, with a negative impact on the molecular and metabolic mechanisms, as well as on the clinical prognosis and post-ICU recovery. An intensely studied mechanism is represented by neuronal Ca^2+^ homeostasis, and by the neuronal metabolic activation. In this situation, it was proven that both biological regulation pathways are modulated by the mitochondria, as it plays a crucial role in the neuronal integrity under the conditions of an accelerated Ca^2+^ dynamic. Recent studies have highlighted a series of mechanisms responsible for CBD induced Ca^2+^ homeostasis. Ryan et al. carried out a complex study regarding CBD action mechanisms in Ca^2+^ homeostasis in neuronal preparation. They observed a series of neuroprotective properties of CBD in neuroblastoma cell lines treated with mitochondrial toxins. Moreover, they showed an important decrease in redox activity and an adequate modulation of Ca^2+^ homeostasis [107]. 

Cannabinoids can also prove useful in the management of pain triggered by inflammation. Recent studies carried out on oncological patients have shown benefic effects on both inflammatory and neuropathic pain. Further molecular implications of cannabinoids, such as decrease in the biosynthesis of pro-inflammatory mediators, as well as modulation of the immune response in in vitro and in vivo studies (Table 2) have been discussed in the literature. Furthermore, other studies on the CB1 expression and its link with the mitochondrial functionality have shown that free radical expression is decreased, while mitochondrial membrane potential is increased [100,101,102,103,104,105,106,107,108,109,110].

## 7. MicroRNAs Expression in Sepsis-Induced by Cannabinoid Signaling System

Another important molecular segment involved in the modulation of the immune response and in the inflammation cascade is represented by the expression of microRNAs [124]. The specific molecular activity of microRNAs in sepsis is complex, with numerous interactions being observed between TLRs and a series of other specific biological signals, such as nuclear factor kappa-light-chain-enhancer of activated B cells (NF-κB), autophagy mechanisms, and apoptosis. Basically, the activity of TLRs is mediated in numerous cases by the expression of microRNAs, with the further modulation of molecular and biological mechanisms for the production of inflammatory mediators. From a biochemical point of view, the microRNAs are non-coding single-stranded RNAs that contain between 19 and 25 nucleotides [125]. Their biosynthesis begins in the cell nucleus through the action of RNA polymerase II on the specific microRNA genes. Following these reactions, the first forms of microRNAs are obtained, (pri-microRNA) that in the end lead to the formation of mature microRNA species [125,126]. The mature species will then be released from the cell as exosomes, apoptotic bodies, or high-density lipoproteins, becoming one of the inter-cellular communication pathways, as well as a pathway for the modulation of specific biochemical and biological processes [127,128,129,130,131,132,133,134,135,136,137,138,139,140,141,142,143,144,145,146,147,148]. These epigenetic mechanisms are also involved in the modulation of the cannabinoid system. Moreover recent studies have proven the existence of certain links between the THC/CBD activity and the response of the CB1 and CB2 receptors by modulating the expression of microRNAs [148,149,150,151,152,153,154,155,156,157].

Juknat et al. have carried out a study in the interactions between CB1 and CB2 receptors with microRNAs following Δ9-THC and CBD activation. For simulating the proinflammatory conditions, they stimulated the BV-2 microglial cells with lipopolysaccharide (LPS), and afterwards, analyzed the effects induced by Δ9-THC on the microRNA expression. Following this study, they reported a significant increase in the expression of microRNA-21, microRNA-146a, and microRNA-155, tightly linked with the biochemical pathways of TLRs and NF-κB. Regarding the CBD activity, they observed a decrease in microRNA-146a and microRNA-155 expressions, as well as an increase in the expression of microRNA-34a [124]. A similar study carried out by Yang et al. presented a decrease in the expression of microRNA-17, microRNA-92, microRNA-421, and microRNA-374b, induced by the action of Δ9-THC [110,111,112,113,114,115,116,117,118,119,120,121,122,123,124,125,126,127,128,129,130].

Recent studies have shown that Δ9-THC modulates and reduces cytokine biosynthesis mediated by T-cells (Th1), as well as the expression of TNF-α and IFN-γ mediated by microRNAs [130,131,132,133,134,135,136,137,138,139,140,141,142,143,144,145,146,147,148,149,150,151,152,153,154,155,156,157,158]. Rao et al. studied the effects induced by Δ9-THC on the inflammatory profile and on the activity of *Staphylococcal enterotoxin* B (SEB). They showed a 100% increase in survival rate in mice that received Δ9-THC treatment, in contrast with the control group, where the mortality was 100%. Regarding the expression of microRNAs, they reported changes in the activity of microRNA-17-92 and microRNA-18a. Moreover, Rao et al. highlighted the potent anti-inflammatory effects of Δ9-THC, and their ability to modulate T-regulatory cells [159]. Chiarlone et al. also reported the involvement of let-7d in the biochemical pathways activating the CB1 receptors [16]. Al-Cghezi et al. investigated the effects on the decrease of neuroinflammation induced by Δ9-THC and CBD in the context of multiple sclerosis. In the experimental study they carried out, they reported a decrease in neuroinflammation through the inhibition of Th17 and Th1 cells’ activity. The combination of Δ9-THC and CBD led to a decrease in CD4^+^ T cells’ activity, and to the decrease in IL-1β, FoxP3, and STAT5b concentrations. Last but not least, they observed a decreased expression for microRNA-21a-5p, microRNA-122-5p, microRNA-31-5p, microRNA-14a-5p, microRNA-150-5p, microRNA-27b-5p, and microRNA-155-5p, and an increase in the levels of microRNA-706-5p and microRNA-7116. The conclusion of their study was that by combining Δ9-THC and CBD, one can change the activity of the microRNAs responsible for the augmentation of the inflammatory mediators’ biosynthesis, therefore leading to a reduction in the inflammatory profile [135,136,137,138,139,140,141,142,143,144,145,146,147,148,149,150,151,152,153,154,155,156,157,158,159,160,161,162,163,164,165].

In a recent study, Sido et al., have shown that after administering THC (20 mg/kg) to C57BL/6 mice leads to a modified expression of the biochemical pathways involved in immune modulation and inflammatory profile. This research group has reported a decrease expression of microRNA-21 that directly lead to a slower immune differentiation of Th17 cells. Increased levels of microRNA-29b have been related to the inhibition of IFN-γ expression [166]. Multiple sclerosis has emerged as a widely studied area of research regarding beneficial effects of THC/CBD in the medical field. Al-Ghezi et al., have carried out a study on the effects induced by THC and CBD (10 mg THC and 10 mg CBD), on the neuronal inflammation specific for multiple sclerosis. Following this study the group has proven a decrease in the expression of microRNA-27b-5p, microRNA-155-5p, microRNA-150-5p, microRNA-146a-5p, microRNA-122-5p, microRNA-21a-5p, microRNA-31-5p, and an increase in the expression of microRNA-706-5p and microRNA-7116 [167]. These changes have been attained by administering CBD/THC on CD4^+^ T cells. Interesting is the fact that microRNAs also modulate a series of biochemical pathways responsible for the inflammatory response. Key elements in the modulation of inflammation are specific molecules such as SOCS1 and FOXP3 [167]. Al-Ghezi et al., have shown that THC + CBD decrease neuroinflammation and that this effect is related to the expression modulation of microRNAs, modulation induced by THC/CBD in the brain [167].

## 8. Immune System Expression-Induced by Cannabinoid Signaling System

The literature states that CB1 receptor is expressed predominantly in the central and peripheral nervous system, while CB2 receptor is mostly found in the immune cells playing a central role in the modulation of the immune response and of the pro- and anti-inflammatory processes. Lehmann et al. have carried out a study regarding the impact of CB2 in the leukocyte activation in two experimental sepsis models. The study was based on four groups of animal models: (a) Control group, (b) LPS group, (c) LPS + CB2 receptor agonist group (HU308, 2.5 mg/kg), and (d) LPS + CB2 receptor agonist group (AM630, 2.5 mg/kg). Following this study they have shown a decrease in leukocyte adhesion capacity after the administration of HU308, as well as a significant decrease of inflammatory mediators after the administration of 10 mg/kg of HU308. The group has concluded that the administration of certain molecules capable of altering biochemical pathways specific for CB2 reduces both leukocyte activation in sepsis and the release of inflammatory mediators [168]. 

**Figure 3:**
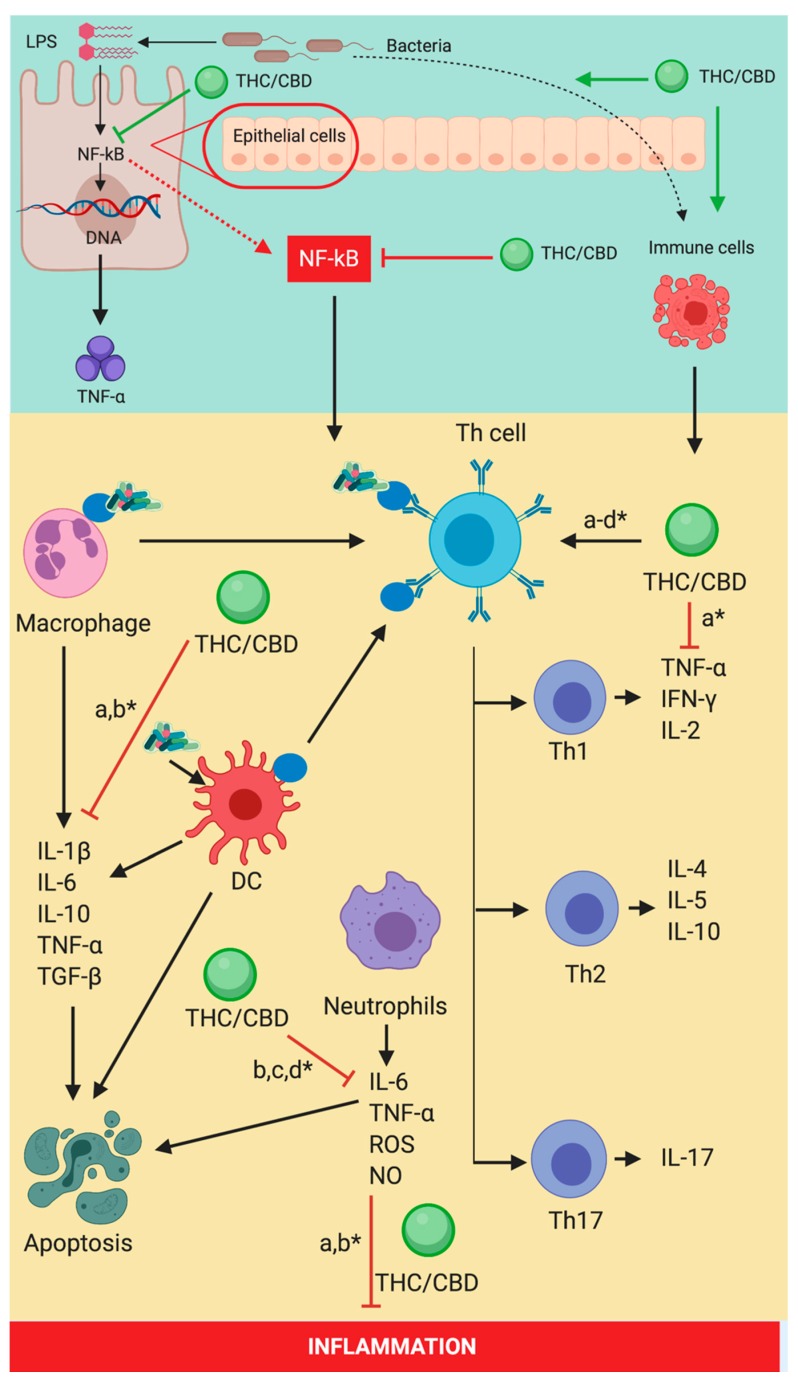
Implications of THC/CBD on the immune response modulation in infections. THC/CBD is responsible for the inhibition of TNF-α, NF-kB and IFN-γ (a* [177]) production. This process takes place through the initial inhibition of Th1 and through the decrease in the expressions of IL-6, TNF-α, and IL-1β by modulating the activity of macrophages (a,b* [177,178]), neutrophils (a,b* [177,178]) and Th cells. THC/CBD: tetrahydrocannabinol/cannabidiol; IFN-γ: interferon gamma; TGF-β: transforming growth factor beta; TNF-α: tumor necrosis factor alpha; IL-1β: interleukin 1 beta; IL2: interleukin 2; IL-4: interleukin 4; IL-5: interleukin 5; IL-6: interleukin 6; IL-10: interleukin 10; ROS: reactive oxygen species; NO: nitrogen oxide; DC: dendritic cells; Th cells: T helper cells.

Cakır et al. have carried out a study on the implications of the CB2 receptor agonist (JWH-133) on the immune response in animal models with induced sepsis. Following their study they have observed an important decrease of histopathological injuries in the brain, lung, liver, and heart of animals that received CB2 receptor agonists (JWH-133). Moreover, they have noticed a decrease in the caspase-3, TNF-α, IL-6, IL-1β, and NF-κB expressions in the same tissues, as well as a decrease in IL-6 and IL-1β serum levels. This research group has also proven an increase in anti-inflammatory cytokines following CB2 receptor agonists [169]. In the context of bacterial invasion the immune system reacts on different levels, the first step being the initial macrophage stimulation through the release of inflammatory mediators. The macrophages then release large amounts of IL-1β, IL-6, and TNF-α, leading to immune activation and disruption of the epithelial barrier. In order to balance out this response anti-inflammatory species such as TGF-β si IL-10 are released. The further release of IL-8 and MCP1 by the macrophages leads to neutrophil stimulation and to their migration at the inflammation site with an over-stimulation of free radicals production, TNF-α and IL-6. The adaptive immune system will also be activated by the dendritic cells (DC) through T-cell modulation and through antigen presentation by the macrophages. This process will lead to T cell differentiation followed by the biosynthesis of specific pro- and anti-inflammatory cytokines and the augmentation of the inflammatory and oxidative cascades (Figure 3) [170,171,172,173,174,175,176,177,178,179,180].

Together with the development of more and more studies in the area of cannabinoids and on their impact on the immune system a series of effects (both of stimulation and inhibition) have been reported. Recent studies have shown that the administration of cannabinoids can lead to a series of reactions of stimulations involved in the control of the immune system. The specific effects of cannabinoids on the T-cell derived cytokine biosynthesis are still contradictory. The literature entails both studies that show an inhibition of Th1 and Th2 cytokine production, and studies that show the opposite. However, recent results have proven that cannabinoids are responsible for a decrease in the expression of certain pro-inflammatory cytokines such as IL-6, IL-12, IL-1, IL-2, and TNF-α. Another important aspect has been presented by Newton et al.; their study reported an increase in Th2 anti-inflammatory species following stimulation with cannabinoids, which is an important response of the humoral immunity [170]. Another similar study shows and increase in the TGF-β expression following cannabinoids [171].

Moreover, THC and CBD have different effects on Th cells. By inhibiting the biosynthesis of Th1 derived pro-inflammatory cytokine and by stimulating the production of anti-inflammatory species by modulating Th2 one can obtain an important therapeutic weapon for the modulation of the immune response and for the reduction of the inflammatory response in certain pathologies. It has also been proven that THC or CBD influence B cells. B cells have an increased expression of CB2 receptor this impacting on the number of B lymphocytes, as well as on the expressions of IgG, IgM, C3 and C4 complement. [172]. Macrophages play an important role in the modulation of the innate immune system and adaptive immune system. Apart from their specific role of antigen presentation for T cells and phagocytosis, macrophages are responsible for the production of certain pro-inflammatory cytokines such as IL-1, IL-6, and TNF-α, and free oxygen and nitrogen radicals. Numerous studies have shown a decreased expression of these cytokines induced by cannabinoids, therefore proving their role in the reduction of the inflammatory profile in certain pathologies [173,174,175]. Recently it was also proven the ability of cannabinoids to lower the expression of IL-6 and NO-induced by LPS [176,177,178,179,180].

## 9. Conclusions

Regarding the link between the endocannabinoid signaling system and the immune and inflammatory changes in the context of sepsis, one can observe numerous important molecular interactions. This is especially due to the increased interaction between CBD receptors and a series of cells representative of the immune system, such as monocytes, leukocytes, polymorphonuclear cells, natural killer cells, and CD4^+^ and CD8^+^ lymphocytes. CB1 and CB2 receptors intervene in numerous processes responsible for inflammation, redox activity, cellular metabolism, and mitochondrial activity. One of the major objectives of this review was to merge the results presented by other studies on the impact of cannabinoids on the main mechanisms involved in sepsis, such as inflammation, redox activity, and the immune expression. Moreover, this article could open new research paths, considering the need for focusing on specific molecular mechanisms in sepsis, as well as on the implications of cannabinoids and of the endocannabinoid signaling system. This article merges the results obtained in the above-mentioned studies from the literature regarding the impact of cannabinoids on the inflammatory profile and on the immune system. The cited studies were carried out either in vitro or in vivo, both on cell lines and on animal models. However, this review article does not recommend using these compounds in the clinical practice without their approval by qualified institutions, or before they are included in current clinical protocols. If we were to summarize it in a molecular model, CBD/THC is involved in most of the biological processed leading to a change in the microRNAs expression and to a blockage in the excessive production of pro-inflammatory cytokines resulting in and immune modulation responsible for a more adequate molecular response in septic patients.

Further research directions aim at validating the pharmacological action of cannabinoids in critically ill patients, with the search for clearer results on the molecular and cellular pathways, as well as on the clinical impact and prognosis. 

## Figures and Tables

**Table 1:** Endocannabinoid signaling system as a potential therapeutic target intervention.

Disorder	Observations	Reference
Pain	CB1 receptor agonists have a nociceptive action on the interneurons in the spinal cord, CB2 acts directly on reducing inflammation, and the CB2 receptor was shown to have an increased immunomodulatory response.	[63]
Cancer	The following effects have been reported: Anti-inflammatory, anti-proliferative, pro-apoptotic, anti-invasive, and anti-metastatic.	[50,51,64]
Hepatic metabolism	Directly acts on the modulation of the hepatic metabolism through gluconeogenesis and lipogenesis, and the CB2 receptor has a protective action on the phenomenon induced by ischemia reperfusion injury.	[47,49,65,66,67]
Gastrointestinal system	CB1 and CB2 receptors inhibit the pro-inflammatory and pro-oxidative activities specifically for the colon.	[49,65,68]
Cardiovascular system	CB2 receptor reduces inflammation specifically related to atheromatous plaques and reduces thrombosis risk; CB1 activates AMP-activated protein kinase (AMPK), reduces insulin resistance, and mimics all of the effects that encompass ischemia-reperfusion injury (IR).	[40,68,69,70]
Immune system/inflammation response	Reduces iNOS activity, reduces IL-6 expression; reduces TNF-α and IL-1β expression; reduces specific inflammation of ARDS/ALI; modulates and reduces the activity of TNF-α and COX-2 in the context of LPS-induced inflammation; inhibits neutrophil chemotaxis; and modulates the expression of IFN-γ, leading to the decrease of IL-2 expression.	[53,71,72,73,74,75]

ALI: acute lung injury; AMPK: AMP-activated protein kinase; ARDS: acute respiratory distress syndrome; CB1: cannabinoid receptor 1; CB2: cannabinoid receptor 2; COX-2: cyclooxigenase 2; IFN-γ: interferon gamma; IL-1β: interleukin 1 beta; IL-2: interleukin 2; IL-6: interleukin-6; iNOS: NO synthase protein; IR: ischemia-reperfusion injury; LPS: lipopolysachharide; TNF-α: tumor necrosis factor alpha.

**Table 2:** Cannabinoids implications on the inflammatory profile and on the immune and redox response.

Cannabinoid	Observations	References
**CBD** **in vitro and in vivo studies**	- ↑analgesic effects by FAAH inhibition and ↑AEA expression; - ↑inflammation response modulation by TRPA1 as an agonist; - By PPARs ↑and ↓in pro-inflammatory expression is obtained; - ↓the intensity of neuropathic pain by PPARs; - ↑antioxidant effects that lead to ↑protection against redox systems for nerve fibers; - ↑anti-proliferative effects on T-cells cellular line; - ↓antigen expression on T cells during major infections; - ↓CD4+ response in mouse autoimmune myocarditis; - ↓CD3+ response in mouse autoimmune myocarditis; - ↓myocardial dysfunction in mouse autoimmune myocarditis; - ↓leukocyte migration on endothelial cells line; - ↑antioxidant expression on J774 cells-LPS stimulus; - ↓NO biosynthesis on J774 cells-LPS stimulus; - ↓IL-6 production on J774 cells-LPS stimulus; - ↓COX-2 expression on J774 cells-LPS stimulus; - ↓inflammation and cytokine production on alveolar macrophages;	[108,109,110,111,112,113,114,115,116,117,118,119,120,121,122]
**THC** **in vivo and in vitro studies**	- Through stimulation CB2 ↓pain-related inflammation - modulate the serotonin biochemical pathways; - TRPA1 activation modulate the inflammatory response; - ↓inflammation and neuropathic pain through modulation of PPARs; - ↓T cell expression and proliferation on T cells—cellular line; - ↓IL-2 biosynthesis on T cells—cellular line; - ↓IFN-γ biosynthesis on T cells—cellular line; - ↓intestinal tissue injury on murine acute graft versus host disease (GVHD) model; - ↓mortality on murine acute GVHD model; - Prevent weight loss on murine acute GVHD model;	[105,106,107,108,109,110,111,112,113,114,115,116,117,118,119,120,121,122,123]

FAAH: fatty-acid amide hydrolase; AEA: anandamide; TRPA1: transient receptor potential cation channel, subfamily A, member 1; PPARs: peroxisome proliferator-activated receptors; CD4^+^: cluster of differentiation 4; CD3^+^: cluster of differentiation 3; LPS: lipopolysachharide; NO: nitric oxide; IL-6: interleukin 6; IL-2: interleukin 2; COX-2: cyclooxigenase 2; CB2: cannabinoid receptor 2; IFN-γ: interferon gamma; GVHD model: models of graft-versus-host disease; ↓ - increase; ↑ - decrease.
